# A Program to Improve Reach Estimation and Reduce Fall Risk in the Elderly

**DOI:** 10.3390/geriatrics1020014

**Published:** 2016-06-06

**Authors:** Carl Gabbard, Kristyn Robinson, Ashley Fox

**Affiliations:** Department of Health & Kinesiology, Texas A&M University, TAMU 4243, College Station, TX 77843, USA; kristynlrob@email.tamu.edu (K.R.); ashfox@tamu.edu (A.F.)

**Keywords:** mental representation, mental imagery, motor imagery, falls

## Abstract

Contemporary research findings indicate that in older persons (typically 64 > years) there are functional decrements in the ability to mentally represent and effectively plan motor actions. Actions, if poorly planned, can result in falling, a major health concern for the elderly. Whereas a number of factors may contribute to falls, over- or underestimation of reach abilities may lead to loss of postural control (balance) and pose a higher risk of falling. Our intent with this paper was to provide: (1) a brief background of the problem, (2) suggest strategies for mental (motor) imagery practice in the context of reach planning, and (3) describe general guidelines and a sample practice format of a training program for clinical use. Mental (motor) imagery practice of reach planning has potential for improving motor performance in reach-related everyday activities and reducing the risk of falls in older persons.

## 1. Introduction

A recent report suggests that every 17 s an older adult is treated for fall-related injuries, and in the next 30 min, an older adult will die from fall-related injuries [1]. The Centers for Disease Control and Prevention [2] report that “one out of three older adults (those aged 65 or older) fall each year”. Furthermore, falls contribute to the highest number of unintentional injuries experienced by individuals aged 65 years and older. There is little debate that falling has significant implications for quality of life and daily living among the elderly. 

While the cause of falling is complex (e.g., disease, medications, visual impairment, balance, environmental factors), recent findings suggest that one of the underlying problems with older adults is difficulty in mentally representing and planning intended actions [3,4,5]. Mental representations are internal cognitive constructs that represent external reality. That information, along with one’s perceived physical abilities, is used to plan and execute movements. The focus of this paper is on reaching actions. While reaching, an individual is likely to consider many questions. Some of these inquires include, “Is the object within or beyond my reachability?” or “Can I reach the object without losing my balance and perhaps fall?” 

In regard to the possible link between reach actions and falls, Gabbard *et al.* [6] and Noël *et al.* [7] reported that overestimation of action capabilities in the context of reaching was a common observation among older adults. Both studies and a recent review by Gabbard [8] noted how overestimation of actions could be a contributing factor in fall risk, especially among the elderly. Furthermore, another review study that looked at the circumstances of falls in elderly people reported that incorrect transfer or shift of body weight was the most frequent cause of falling (41%) [1]. A specific example of this was leaning too far from one’s base of support, an observation commonly seen during under- or overestimation in reach situations.

The purpose of this brief communition is to highlight the link between one’s reach planning and risk of falling and provide suggests for intervention with populations at risk.

## 2. Mental Representation and Reach Planning

A process frequently mentioned in parallel with mental representation is mental imagery. Furthermore, motor imagery, also known as kinesthetic imagery, is mental imagery that is more specific to a motor context. Motor imagery is an active cognitive process during which the representation of a specific action is internally reproduced (simulated) in working memory without any overt motor output [9]. More importantly, researchers suggest that motor imagery provides a window into the process of action representation by reflecting an internal action representation [10,11]. The ability to predict an intended action outcome and its consequences is important in action planning. It has also been hypothesized that motor imagery plays an important role in predicting actions [12,13,14,15]. For example, when planning an intended action, using imagery to consider important timing and biomechanical information helps predict the movement’s sensory consequences. Imagery allows us to generate specific predictions based upon past experience. Effective imagery also allows us to answer ‘what if’ questions by making the likely consequences of a specific action explicit and accessible. 

## 3. Mental Representation in Older Adults

The consensus of research indicates that in older adults (>64 years) the ability to mentally represent motor actions declines signficantly [3,5,16,17,18,19,20,21]. For example, using a mental chronometry paradigm with imagined and executed arm pointing action in young and older adults, Personnier and colleagues [17] found that although older adults displayed the ability to mentally represent action via use of motor imagery, this ability progressively deteriorated with advancing age. That is, there was less isochrony between what they imagined and their executed movements. Those findings also hinted at the likelihood of a weaker formulation of internal models of action. Testing the ability to mentally simulate/plan a complex sequential action of the whole body (*i.e.*, rising from the floor), Saimpont *et al.* [18] reported the elderly experienced significant difficulties compared to young adults’ accuracy in action sequence and reaction time. In a more recent review of this body of research pertaining to individuals 55 years and older, Saimpont and colleagues [20] concluded that motor imagery accuracy for simple/familiar upper-limb movements appears well preserved with aging, whereas there appears to be an aging effect with upper-limb actions with unusual biomechanical constraints. Furthermore, in a more recent study conducted by Saimpont and colleagues [21], motor imagery ability in younger and older adults was compared by administering the subjects three tests: a kinesthetic and visual imagery questionnaire, a finger-thumb opposition task, and a chronometric task. Results revealed that performance was similar between age groups for the questionnaire and chronometric task, but the younger group displayed a higher level of accuracy with the finger-thumb opposition test. In addition, there was significant variability in the performace within groups, suggesting the importance of considering each person individually in their motor imagery abilities. In support of the observation of variability among the elderly, Kalicinski and Raab [22] found that elder adults show no age-related alterations of motor imagery for familiar movements. However, in movements that are unfamiliar, there may be weakness and instability. One common mediator in cognitive processing, including mental representation and imagery use, is working memory, which typically shows a decline with advancing age. Schott [23] found that with individuals older than 70 years, working memory was a significant mediator in those individual’s ability to use motor imagery. Interestingly, the group of 20–30 year-olds were comparable to a group of 60–69 year-olds. 

In summary, it would appear that older persons have difficulty estimating possible movement outcomes. This weakness in motor planning results in a dissociation between perception and action, a condition that may promote risky motor planning. With the aforementioned information on a general decline in motor imagery ability with advancing age, one should keep in mind the likelihood of significant variability between individuals when designing and implementing training programs. 

## 4. Motor Imagery Training

Despite the literature being sparse in regard to the effectiveness of imagery training in the context of reach, studies using other actions are promising. For example, motor imagery has been documented as an effective training and therapy tool for recovery from brain injury [24] stroke [25,26], and other neuromotor impairments [27,28]. In healthy individuals, research findings are also supportive of the benefits of motor imagery training for planning actions [29,30,31,32]. The literature suggests that one of the key agents in movement recovery is use of motor imagery to stimulate otherwise non-active or impaired neural pathways. Wohldmann and colleagues [33] reported evidence to support the hypothesis that mental practice strengthens abstract mental representation that does not involve specific effectors. That is, such practice strengthens ‘central’ features of the representation as well as representation of specific body part processes. It is also important to note that of either visual or motor (kinesthetic) imagery, motor imagery, as proposed here, has shown to be more effective in corticomotor stimulation [34,35]. 

## 5. General Strategies for Motor Imagery Practice

Whereas the information on clinical strategies to improve mental imagery among the elderly is sparse, information derived from training studies among brain and stroke patients may be applicable [36,37]. Those strategies include (as suggested in part by Gabbard and Fox [38]:

**Clear and effective script of instructions:** The script of instructions should be used in early practice phases and needs to be as specific as possible. For example: “Watch and feel your hand and fingers reaching and grasping the mug. How will you grasp the mug and how fast will you move as not to spill its contents?” 

**Goal-setting:** This is an important element of practice and a strategy that can be induced via the script, verbally, or in combination as reinforcement. 

**First person internalizing:** That is, focus on the self as the mental image of the intended action. This state is the first component in the actor-object dyad. Part of this dyad is an understanding of one’s physical capabilities and potential consequences. Ask the participant to consider the consequences if he or she were to misplan (over- or underestimate) the event. What will you fall into? Do you feel confident in your ability to gain stability if you have to adjust your position quickly? 

**Concentrate on the effectors:** This is the specific part of the self (e.g., arm, hand, fingers) that is linked with the target. For example, focus on the arm/hand when intending to reach and grasp an object. 

**Focus on the visual cues (target/object/goal):** Concentrate on the end point of the intended action—the target. For example, where does your hand need to be to grasp the object securely? Auditory cues in the form of a metronome have been effective with tasks such as aiming at a target when time is a factor. 

**Reinforcement on kinesthetically ‘feeling’ execution of movement:** Research clearly indicates that ‘feeling’ rather than just seeing oneself perform the action promotes better mental representation for movement via internalization. 

**Combine physiotherapy (physical practice) with mental practice:** By practicing actual movements, participants gain a better understanding of their physical capabilities and develop movement endurance. Physical practice affords the opportunity for one to test his/her capabilities. 

**Progress from simple to more complex actions:** Do this when practicing both imagined and actual execution movements. 

**Practice 15–60 min, three times/week for four weeks:** This has been one of the most commonly reported practice durations for imagery training with a variety of patient impairments. Obviously, this should be viewed as an approximate benchmark with the progression principle strictly applied. These figures come from our review of published effective MI intervention programs [24,25,26,27,28,29,30,31,32].

## 6. General Mental Practice Guidelines

One of the first considerations in creating such a training program, especially with the elderly, is safety. Especially in this context, measures to prevent falls need to be taken (use of human support, support devices, mats, *etc.*). Then, one should consider the suggested strategies provided earlier. For example, ‘provide clear instructions,’ ‘have the individual visualize and *feel* the movements,’ ‘focus on the target and distance to you,’ ‘combine physical practice with mental practice (motor imagery),’ and ‘progress from simple to more complex actions,’ as described previously. 

Using the general day/time recommendations derived from our literature review noted earlier, three times a week [MWF] starting with 15 min and gradually increasing to about one hour, we tested the practicality and safety of this specific motor imagery training (for reach actions) with a small group of healthy community dwelling senior citizens (aged 65#x2013;81 years). Each training session began with a relaxation segment, followed by a detailed explanation/reminder of how the training will be organized, as well as how to use motor imagery (*feeling* the movement) throughout the entirety of the session, which lasted four weeks. We do wish to note that by no means was the pilot an experimental comparison of various regimens. No pre- or post-test data were collected for this project; we were only interested in practicality and safety of this specific motor imagery training (for reach actions) with older persons. 

**Variations** (the goal is variability of movement in a variety of everyday activity situations with the focus on mentally planning reach actions via use of imagery):

Reach for: different *everyday objects*: cup, newspaper, book
Reach from: different *body positions*: seated, standing, leaning (bending) over
  a variety of *surfaces*: flat, moving upstairs, moving downstairs
  different *levels*: knee level, waist, overhead, eye-level, and floor
  various *angles*: midline, right, left, *etc.*
  different *distances* to the object/target. Before starting this, determine the individual’s actual
    maximum (arm extended) reach. Then place objects around their maximum reach
    point to assess their estimation.

Using dominant and non-dominant limbs.

### Sample Instructions

First session while seated: “Today you will be judging whether or not you can reach objects and then actually reach for objects placed at various locations on the table.” 

“Remember focus and ‘feel’ (sense) moving your body—hand/arm/trunk, legs, without actually moving. For example, Can you reach the object without leaning over? Without stepping forward? Is the object within or out of your comfortable *reach?”*

While seated and standing: “What would be a consequence if you over- or under-reached?” 

## 7. Conclusions

Research findings present a relatively clear indication that, with the elderly, the ability to effectively plan intended actions becomes increasingly more difficult. As in the context of reach, ineffectiveness could pose a higher risk of falling. Imagery training, specifically motor imagery training, has been effective in improving motor behavior and action planning among different populations. Therefore, as this paper suggests, imagery training has the potential to be useful in significantly reducing the risk of falling in the elderly. 
